# In vitro reconstitution of calcium-dependent recruitment of the human ESCRT machinery in lysosomal membrane repair

**DOI:** 10.1073/pnas.2205590119

**Published:** 2022-08-22

**Authors:** Sankalp Shukla, Kevin P. Larsen, Chenxi Ou, Kevin Rose, James H. Hurley

**Affiliations:** ^a^Department of Molecular and Cell Biology, University of California, Berkeley, CA 94720;; ^b^California Institute for Quantitative Biosciences, University of California, Berkeley, CA 94720;; ^c^Helen Wills Neuroscience Institute, University of California, Berkeley, CA 94720

**Keywords:** membrane biology, membrane remodeling, membrane repair, in vitro reconstitution, neurodegeneration

## Abstract

The endosomal sorting complex required for transport (ESCRT) machinery is centrally involved in the repair of damage to both the plasma and lysosome membranes. ESCRT recruitment to sites of damage occurs on a fast time scale, and Ca^2+^ has been proposed to play a key signaling role in the process. Here, we show that the Ca^2+^-binding regulatory protein ALG-2 binds directly to negatively charged membranes in a Ca^2+^-dependent manner. Next, by monitoring the colocalization of ALIX with ALG-2 on negatively charged membranes, we show that ALG-2 recruits ALIX to the membrane. Furthermore, we show that ALIX recruitment to the membrane orchestrates the downstream assembly of late-acting CHMP4B, CHMP3, and CHMP2A subunits along with the AAA^+^ ATPase VPS4B. Finally, we show that ALG-2 can also recruit the ESCRT-III machinery to the membrane via the canonical ESCRT-I/II pathway. Our reconstitution experiments delineate the minimal sets of components needed to assemble the entire membrane repair machinery and open an avenue for the mechanistic understanding of endolysosomal membrane repair.

## Significance

One of the ways by which protein aggregates can propagate and lead to the progression of a neurodegenerative disease is by damaging the membrane that is destined to degrade these misfolded aggregates. The ESCRT machinery has been implicated in sealing these damaged membranes, and the nature of the membrane recruitment trigger signal for this machinery is a major open question. Here, we show in vitro that ALG-2 alone is sufficient to bring the ESCRT machinery to membranes in a Ca^2+^-dependent manner.

The endolysosomal system is susceptible to damage by a number of factors, including protein aggregates, lysosomotropic compounds, reactive oxygen species, and lipid metabolites (1). Endosomal escape mediates the entry into the cytosol of many viruses (2), therapeutic agents (3, 4), and the transmission of prion-like molecular aggregates (5). Escape is counteracted by the protective effects of lysosomal membrane repair (6), which is carried out by the endosomal sorting complex required for transport (ESCRT) membrane sealing machinery. The ESCRTs are a conserved membrane scission and sealing machinery consisting of about 30 proteins in humans (7, 8). In particular, ALIX, ESCRT-I, and the ESCRT-III subunits CHMP6, CHMP2A, and CHMP2B have been implicated in cytoprotective lysosomal membrane repair events (6, 9, 10). ESCRTs are also involved in the repair of the plasma membrane (11), where extracellular Ca^2+^ influx has been shown to trigger the subsequent recruitment of ESCRT-III machinery to the damaged plasma membrane site (12). The eventual closure of damage-induced holes in the plasma membrane is achieved by membrane budding and shedding vesicles toward the extracellular space by ESCRT-III machinery (11).

Lysosomes have nearly a 5,000-fold higher concentration of Ca^2+^ (∼0.5 mM) compared to that in cytosol (∼100 nM) (13, 14). Therefore, damage to the endolysosomal membrane locally increases Ca^2+^ concentration near the site of damage. Increased local Ca^2+^ efflux into the cytosol has been proposed to be a trigger for the recruitment of endolysosomal membrane repair machinery (9). The Ca^2+^ binding protein ALG-2 coaccumulates with ALIX upon damage and has been proposed to have an upstream role in the endolysosomal membrane repair sequence (9). ALG-2 contains five serially repetitive EF-hand structures and is the most conserved protein among the penta-EF-hand (PEF) family (15). Upon binding to Ca^2+^, ALG-2 undergoes conformational changes, rendering it amenable to bind to proline-rich proteins such as ALIX (16). Therefore, ALG-2 is a prominent candidate to trigger the recruitment of ESCRT-III machinery, and therefore endolysosomal membrane repair, in response to increased local Ca^2+^ concentration around a damaged endolysosome. However, whether ALG-2 is sufficient to trigger the membrane recruitment of repair machinery at the site of damage is unclear (17).

Here, we investigated the mechanism directly through in vitro reconstitution in a completely defined system of purified proteins and synthetic lipids. In vitro reconstitution is a powerful tool to determine the sufficiency of a biochemical factor, which we applied here to probe whether ALG-2 is sufficient to recruit downstream components of the endolysosomal membrane repair pathway. We used a giant unilamellar vesicle (GUV) reconstitution system to take advantage of fluorescence microscopy for our reconstitution experiments. We first showed that ALG-2 can be recruited to the negatively charged membranes without the need for an upstream adaptor protein. By using a Ca^2+^-binding–deficient mutant of ALG-2, we confirmed that ALG-2 membrane recruitment is mediated by Ca^2+^. By monitoring the colocalization of ALIX with ALG-2 on the negatively charged membrane, we confirmed the upstream role of ALG-2 in bringing ALIX to the membrane. We showed the complete downstream recruitment of human ESCRT-III machinery vis-à-vis CHMP4B, CHMP2A, and CHMP3 along with the AAA^+^ ATPase VPS4B to the negatively charged membranes in an ALG-2– and Ca^2+^-dependent manner. We demonstrated that ALG-2 also recruits the ESCRT-III machinery via the canonical ESCRT-I/II pathway. Finally, we validated that endolysosomal membrane damage leads to colocalization of ALG-2, ALIX, and ESCRT-I in vivo.

## Results

### ALG-2 Binds to Negatively Charged Membranes in a Ca^2+^-Dependent Manner.

**Fig. 1.**
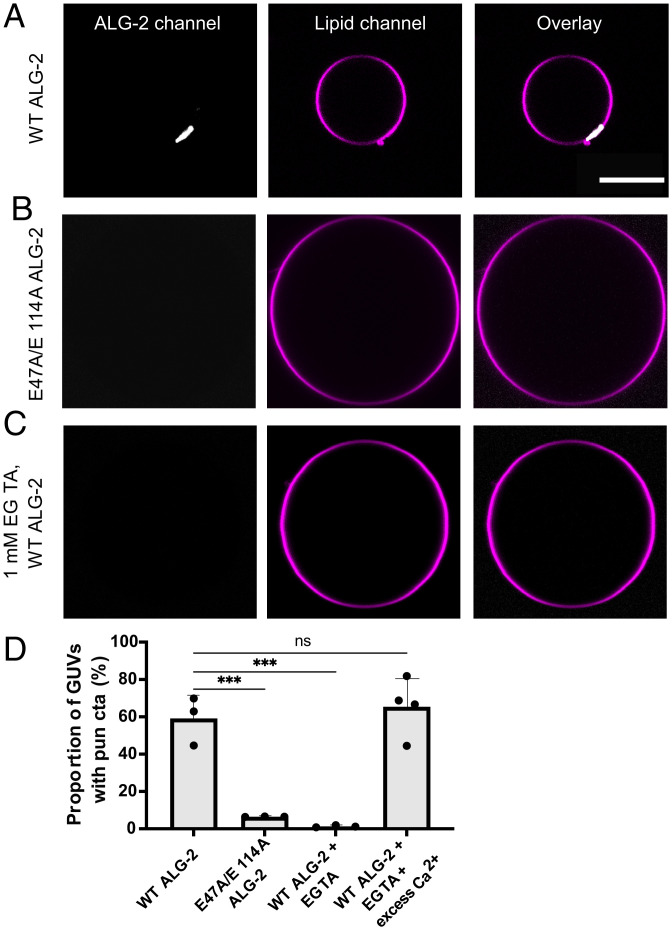
Membrane recruitment of ALG-2 is Ca^2+^ dependent. The GUVs were prepared using the PVA-gel hydration-based protocol described in *Materials and Methods*. ALG-2 was fluorescently labeled using Atto 488 maleimide by introducing a solvent-accessible mutation, A78C in WT ALG-2. (*A*) Atto 488–labeled ALG-2 is recruited to the periphery of the 30% DOPS containing GUVs. The periphery of a GUV was determined by using the fluorescence of the lipid dye (Atto 647NDOPE) added in trace amounts (0.5%) to the lipid mixture used for GUV preparation. (*B*) On replacing the WT ALG-2 with the Ca^2+^-binding–deficient mutant of ALG-2 (E47A/E114A), no detectable ALG-2 fluorescence was observed at the GUV periphery. (*C*) Using an ALG-2 stock incubated with 1 mM EGTA overnight also abrogated the membrane binding of ALG-2. The absence of ALG-2 puncta on GUVs confirms that the membrane recruitment of ALG-2 is calcium dependent. (*D*) The proportion of GUVs that had at least one ALG-2 punctum on their periphery were plotted for fluorescently labeled WT ALG-2 (*n* = 2066 GUVs), E47A/E114A ALG-2 (*n* = 507 GUVs), 1 mM EGTA incubated ALG-2 (*n* = 389 GUVs), and EGTA incubated ALG-2 rescued with excess calcium (*n* = 83 GUVs). The circles on the bar charts represent independent data points and the data are shown as mean ± SD (vertical line). All results are from at least three independent experiments. *P* ≤ 0.003 (***), ns = not significant. (Scale bar, 10 μm.)

We set out to decipher the role of the endolysosomal membrane repair machinery upstream of ESCRT-III recruitment at the site of endolysosome membrane damage. To do this, we used in vitro GUV reconstitution experiments to sequentially test the membrane binding ability of the endolysosomal membrane repair machinery. We incubated GUVs containing 30% 1,2-dioleoyl-sn-glycero-3-phospho-L-serine (DOPS) along with 69.5% 1,2-dioleoyl-sn-glycero-3-phosphocholine (DOPC) and 0.5% Atto 647N dye-labeled 1,2-dioleoyl-sn-glycerol-3-phosphoethanolamine (DOPE) with ALG-2 (Atto 488; 200 nM) for 15 min in reaction buffer. On imaging, we observed ALG-2 puncta on the GUVs (Fig. 1*A*), suggesting that ALG-2 can bind to negatively charged membranes without the need for an upstream membrane anchor. Surprisingly, membrane recruitment of ALG-2 occurred in the absence of externally added Ca^2+^. Furthermore, the external addition of Ca^2+^ to a concentration of 100 μM did not affect the binding of ALG-2 to membranes.

We hypothesized that ALG-2 was purified in the Ca^2+^-bound form and therefore was already in its activated state. To test our hypothesis, we purified a Ca^2+^-binding–deficient mutant (E47A/E114A) (15) of ALG-2 and performed the same membrane-binding experiment. We found that the Ca^2+^-binding–deficient mutant (E47A/E114A) of ALG-2 did not bind 30% DOPS-containing membranes (Fig. 1*B*). Moreover, the addition of 100 μM CaCl_2_ to ALG-2^E47A/E114A^ did not affect its membrane nonbinding behavior. Additionally, we incubated a purified stock of ALG-2 (50 μM) with 1 mM ethylene glycol tetraacetic acid (EGTA) (pH 7.4) overnight. On using the EGTA incubated ALG-2 stock to perform membrane binding experiments, we did not observe ALG-2 puncta on the membrane (Fig. 1*C*). Furthermore, to rescue the membrane binding activity of the EGTA incubated ALG-2 stock, we diluted the stock of ALG-2 incubated with EGTA ([ALG-2] = 50 μM; [EGTA] = 1 mM) to the final concentration of ALG-2 used in the membrane-binding experiments, i.e., 200 nM. At this dilution, the concentration of EGTA is 4 μM. Next, we added excess calcium (100 μM) to convert the calcium-depleted ALG-2 to the calcium-bound (activated) form. This resulted in the rescuing of the membrane-binding ability of ALG-2 (Fig. 1*D*). Together with the results for ALG-2^E47A/E114A^, calcium depletion with EGTA, and final rescue of the membrane binding with excess calcium, we confirmed that Ca^2+^ is likely the trigger for recruiting ALG-2 to the negatively charged membranes.

### ALG-2 Is Necessary for Membrane Recruitment of ALIX.

**Fig. 2.**
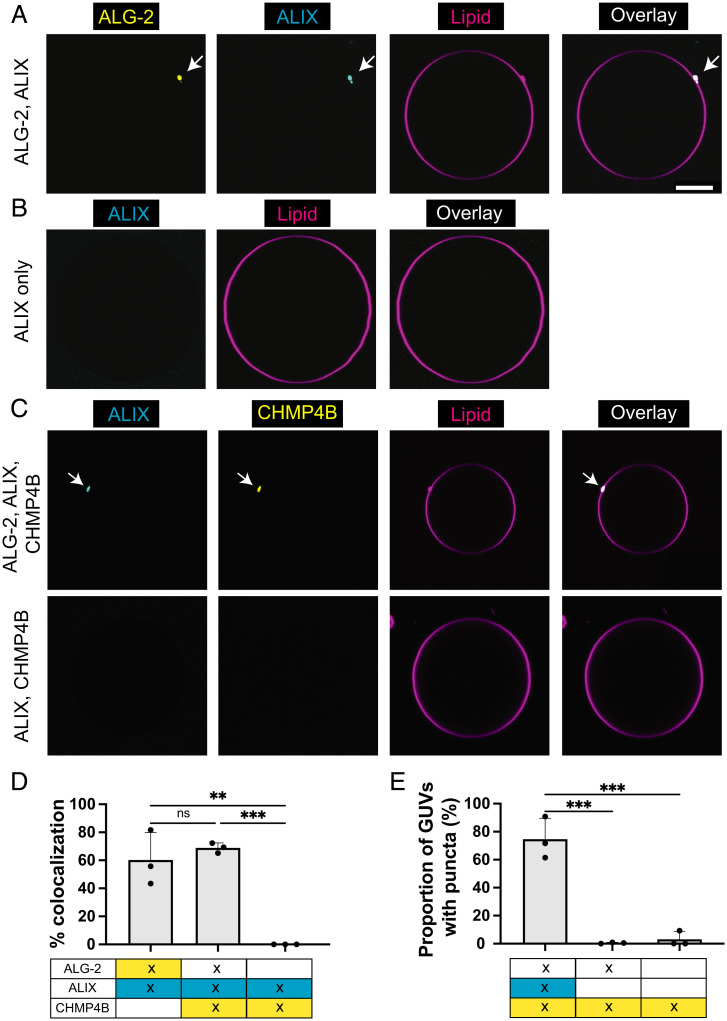
ALG-2 activates and corecruits ALIX to membranes. (*A*) GUVs containing 30% DOPS are incubated with fluorescently labeled ALG-2 (Atto 488; 200 nM) and ALIX (Cy3; 100 nM). We observe a clear colocalization between the membrane-recruited ALG-2 and ALIX. (*B*) In the absence of ALG-2, no ALIX puncta were observed on the membranes. (*C*) (Upper row) Addition of ALG-2 (dark; 200 nM) and ALIX (Cy3; 100 nM) along with CHMP4B (Atto 488; 10 nM) to 30% DOPS containing GUVs results in colocalized puncta of ALIX and CHMP4B. (Lower row) In the absence of ALG-2, there was no observable membrane recruitment of ALIX and CHMP4B onto the membrane. (*D*) Bar chart depicting the colocalization % of the puncta in two different fluorescently labeled protein channels. (*E*) Bar chart depicting the proportion of GUVs that had at least one punctum for the conditions specified underneath each bar chart. Where two labeled proteins were present, the proportion of GUVs with puncta was reported for the most downstream protein (i.e., between ALIX and CHMP4B, the bar chart depicts the results for CHMP4B puncta). At least 300 GUVs from three independent experiments were analyzed for each set of conditions plotted in the bar chart. The circles on the bar plot represent independent data points and the data are shown as mean ± SD (vertical line). *P* < 0.0016 (**), *P* < 0.0008 (***), ns = not significant. (Scale bar, 5 μm.)

ALIX is an ESCRT accessory protein that forms an alternative pathway to that of ESCRT-I/II for the recruitment and activation of ESCRT-III in humans (18). ALIX comprises a Bro1 domain, a V domain, and a proline-rich domain (PRD), and functions as a homodimer (19). Earlier work has shown that ALIX exists in an autoinhibited conformation (20) and requires an upstream factor for its activation. The upstream binding of ALG-2 to the PRD of ALIX in a Ca^2+^-dependent manner has been shown to release the autoinhibition of ALIX and make the Bro1 domain accessible to bind downstream ESCRT-III machinery (21). With this understanding, we incubated ALG-2 (Atto 488; 200 nM) with ALIX (Cy3; 100 nM) in the reaction buffer for 15 min before delivering them to 30% DOPS GUVs. We found that ALIX is recruited to the negatively charged membranes (30% DOPS) only when ALG-2 is present (Fig. 2*A*). In the absence of ALG-2, we did not observe membrane binding of ALIX (Fig. 2*B*). External addition of CaCl_2_ to the ALIX-only sample did not promote membrane binding of ALIX.

Additionally, ALIX was not recruited to the membrane by the Ca^2+^-binding–deficient mutant of ALG-2. The reason for the absence of ALIX puncta on the membrane on replacing wild-type (WT) ALG-2 with Ca^2+^-binding–deficient mutant of ALG-2 could be twofold. First, the interaction between ALIX and ALG-2 has been shown to be mediated by Ca^2+^ (22). Second, based on our earlier observation, ALG-2 membrane interaction is Ca^2+^ mediated. Therefore, even if there is an interaction between ALIX and Ca^2+^-binding–deficient mutant of ALG-2, ALIX will not be recruited to membranes. Furthermore, we checked that ALG-2–dependent ALIX recruitment to the membrane (and conversely, membrane nonbinding of ALIX by Ca^2+^-binding–deficient mutant of ALG-2) existed for labeled as well as dark ALG-2, suggesting that fluorophore labeling of ALG-2 did not interfere with the recruitment of endolysosomal membrane repair machinery (*SI Appendix*, Fig. S1). Therefore, for subsequent experiments, we used dark ALG-2 in conjunction with labeled downstream proteins of the endolysosomal membrane repair machinery to prevent bleed through between imaging channels.

### The Downstream ESCRT-III Machinery Is Recruited to ALG-2/ALIX Puncta.

ALIX has been shown to recruit the downstream ESCRT-III machinery to the membrane upon an upstream cue (23, 24). In the cytosol, ESCRT-III proteins exist in a C-terminal autoinhibitory state (25) that needs to be released for them to be functional (26, 27). Specifically, the Bro1 domain of ALIX interacts with the C-terminal α6 of the CHMP4B (28). CHMP4B (and other ESCRT-III proteins) electrostatically bind to negatively charged membranes through their electropositive amino-terminal residues as well as through the insertion of their N-terminal amphipathic helix (29).

CHMP4B, CHMP2A, and CHMP3 along with VPS4B are the human counterparts of the minimal yeast ESCRT-III machinery needed for the membrane scission (30). We found that under our experimental conditions (reaction buffer and 30% DOPS GUVs), CHMP4B can bind to membranes with no upstream factor at a concentration of 50 nM (*SI Appendix*, Fig. S2). At this concentration, CHMP4B coats the entire membrane surface within minutes after incubation with GUVs. We speculate that the membrane plays a role in the release of autoinhibition of CHMP4B at this concentration, an effect that could be allosteric.

Consistent with earlier experiments (31), we found that CHMP4B is necessary to recruit the downstream heteropolymers of CHMP2A and CHMP3. The addition of 100 nM CHMP2A and CHMP3 each to 50 nM CHMP4B resulted in the recruitment of CHMP2A/3 heteropolymers to GUVs. Neither CHMP2A nor CHMP3 was recruited to 30% DOPS GUVs at a concentration of 100 nM each in the absence of CHMP4B. Therefore, at a concentration of CHMP4B where it can bind to membranes on its own, the downstream ESCRT-III machinery also gets recruited to the membrane.

However, physiologically, ESCRT-III proteins are cytosolic in their resting state and are recruited to membranes only in response to an upstream cue (27). Accordingly, to mimic the physiological machinery, we decreased the concentration of CHMP4B to avoid its self-recruitment to membranes. We found that at a concentration of 10 nM, CHMP4B is no longer recruited to 30% DOPS GUVs in the absence of an upstream binding partner. At this concentration of CHMP4B (10 nM), the addition of ALG-2 (200 nM) and ALIX (100 nM) resulted in the recruitment of CHMP4B to the membrane. The membrane-recruited CHMP4B puncta colocalized with ALG-2/ALIX puncta (Fig. 2*C*, upper row and 2*D*). Additionally, the omission of ALIX prevented the recruitment of CHMP4B to the membrane (Fig. 2*C*, lower row and 2*E*).

### Complete Reconstitution of ESCRT-III Machinery in Response to Ca^2+^ Binding by ALG-2.

It has been shown that AAA^+^ ATPase VPS4 is necessary for membrane scission by virtue of disassembling ESCRT-III polymers on membrane necks (32). The established recycling function of VPS4 depends on the interaction of the MIT domain in VPS4 with MIT-interacting motifs (MIMs) in ESCRT-III subunits (33, 34). Therefore, membrane-recruited upstream ESCRT-III proteins should also recruit VPS4B. To test this in our experiments, we added 100 nM VPS4B and saw its colocalization with 50 nM CHMP4B, 100 nM CHMP2A, and 100 nM CHMP3 on 30% DOPS membranes. In the absence of ESCRT-III proteins, VPS4B was not recruited to membranes even after increasing its concentration to 500 nM (*SI Appendix*, Fig. S3).

**Fig. 3.**
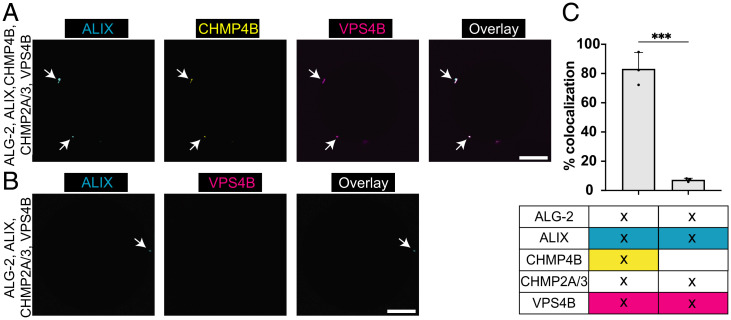
Complete reconstitution of Ca^2+^-triggered endolysosomal membrane repair machinery. (*A*) Fluorescently labeled ALIX (Cy3; 100 nM), CHMP4B (Atto 488; 10 nM), and VPS4B (LD 655; 100 nM) along with ALG-2 (dark; 200 nM), CHMP2A (dark; 100 nM), and CHMP3 (dark; 100 nM) were mixed with 30% DOPS containing GUVs and imaged. The observance of colocalized puncta of the labeled proteins on the periphery of GUVs confirms the recruitment of the entire endolysosomal membrane repair machinery. (*B*) In the absence of CHMP4B, labeled VPS4B was not recruited to the ALIX puncta on the membrane. (*C*) The bar chart depicting the colocalization % of the puncta in two different fluorescently labeled protein channels. At least 100 GUVs from three independent experiments were analyzed for each set of conditions plotted in the bar chart. The circles on the bar plots represent independent data points and the data are shown as mean ± SD (vertical line). *P* = 0.0003 (***). (Scale bar, 10 μm.)

For the complete reconstitution of the endolysosomal membrane repair machinery, we used fluorescently labeled ALIX (Cy3; 100 nM), CHMP4B (Atto 488; 10 nM), and VPS4B (Lumidyne 655 (LD 655); 100 nM). All other proteins involved in the reconstitution machinery, namely, ALG-2 (200 nM), CHMP2A (100 nM), and CHMP3 (100 nM), were unlabeled. All the above-mentioned proteins were mixed in the reaction buffer at the aforementioned concentrations and incubated for 30 min before adding GUVs. The imaging was started 15 min after GUV addition. We found that all the three labeled proteins were colocalized on the GUV surface (Fig. 3*A*). Additionally, the omission of either CHMP4B or CHMP2A/3 resulted in the abrogation of membrane recruitment of VPS4B (Fig. 3*B*). We show the colocalization statistics of these experiments in Fig. 3*C*.

Above, we showed that ALIX is recruited to membranes only in the presence of Ca^2+^-activated ALG-2. We have also shown that VPS4B is recruited to a 30 mol% negatively charged membrane only when CHMP2A and CHMP3 are present. Therefore, the recruitment of VPS4B to membranes is a confirmation that CHMP2A and CHMP3 are also colocalized at the membrane. In conclusion, we demonstrate the complete reconstitution of the ALIX-mediated endolysosomal membrane repair machinery, starting from the Ca^2+^-activated ALG-2 to the AAA^+^ ATPase VPS4B.

### ALG-2 Recruits ESCRT-I Complex to Negatively Charged Membranes.

ESCRT-I, ESCRT-II, and CHMP6 form the canonical pathway for the recruitment of the ESCRT-III machinery (7, 8). Human ESCRT-I is a soluble heterotetramer consisting of TSG101, VPS28, VPS37(A–D) and MVB12 (A, B)/UBAP1/UBA1L/UMAD1. Earlier mutation-based experiments have shown the interaction between the proline-rich region of the TSG101 subunit of ESCRT-I with ALG-2 in a Ca^2+^-dependent manner (35). Also, it has been observed in vivo that membrane repair is completely stalled only upon codepletion of ALIX and TSG101 and not upon individual depletion of either of these proteins (9). When depleted individually, loss of TSG101, but not ALIX, was shown to reduce cell viability (10). These data implied a central role for ESCRT-I and motivated us to ask the question of whether ALG-2 could be the upstream connecting link in recruiting ESCRT-III machinery for membrane repair via ESCRT-I.

**Fig. 4.**
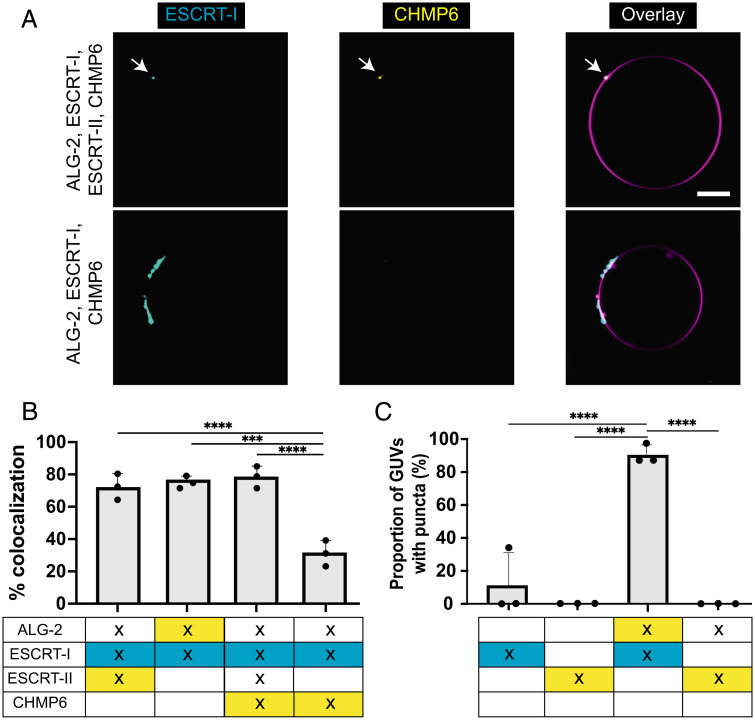
ALG-2–mediated canonical ESCRT-III recruitment pathway. (*A*) (Upper row) GUVs containing 30% DOPS are incubated with ALG-2 (dark; 200 nM), ESCRT-I (Cy3; 50 nM), ESCRT-II (dark; 200 nM), and CHMP6 (Atto 488; 400 nM). Imaging post 15-min of incubation resulted in ESCRT-I, CHMP6 colocalized puncta on the surface of GUVs. This confirmed that the ALG-2 recruits the downstream cascade of ESCRT-III machinery via ESCRT-I and ESCRT-II. (Lower row) On omitting ESCRT-II, the CHMP6 puncta on the membrane were mostly missing. The CHMP6 puncta that were occasionally observed rarely coincided with the ESCRT-I puncta. (*B*) Bar charts for the colocalization % of the puncta observed for membrane-recruited proteins. (*C*) The proportion of GUVs that had at least one punctum for the conditions specified underneath each bar chart. In the scenario where two labeled proteins were present, the proportion of GUVs with puncta was reported for the most downstream protein (i.e., between ALG-2 and ESCRT-I, the bar chart depicts the results for ESCRT-I puncta). At least 390 GUVs for colocalization experiments and 200 GUVs for the proportion of GUVs with puncta from three independent experiments were analyzed for each set of conditions plotted in the bar chart. The circles on the bar charts represent independent data points and the data are shown as mean ± SD (vertical line). *P* = 0.0002 (***), *P* < 0.0001 (****). (Scale bar, 10 μm.)

We incubated fluorescently labeled ALG-2 (Atto 488; 200 nM) with ESCRT-I complex (Cy3; 50 nM) in the reaction buffer for 15 min. Because ESCRT-I can weakly bind to negatively charged membranes through its MABP domain (36), we used a concentration of ESCRT-I that was low enough that it did not bind GUVs on its own (*SI Appendix*, Fig. S4). Next, we added 30% DOPS GUVs to this mixture and imaged them after a 15-min incubation. We found that the ESCRT-I complex colocalized with the ALG-2 puncta on the membrane (Fig. 4*A*, upper row and 4*B*). Under these conditions, we did not see statistically significant membrane recruitment of ESCRT-I in the absence of ALG-2 (Fig. 4*C*).

Next, we asked whether we could reconstitute the canonical pathway of ESCRT-III recruitment downstream of ESCRT-I. Human ESCRT-II is a Y-shaped tetrameric complex comprising two EAP20 subunits and one copy each of EAP30 and EAP45 (37–39). It has been shown that the VPS28 subunit of the ESCRT-I complex interacts with the EAP45 subunit of the ESCRT-II complex (40). Therefore, we incubated fluorescently labeled ESCRT-II (Atto 488; 200 nM) complex with ESCRT-I (Cy3; 50 nM) complex and ALG-2 (dark; 200 nM) for 15 . Thereafter, GUVs were added and imaged after 15 of incubation. We found that both ESCRT-I and ESCRT-II colocalize on the GUVs (Fig. 4*B*). In contrast, we did not observe membrane binding of ESCRT-II in the absence of either ALG-2 or ESCRT-I (Fig. 4*C*).

Furthermore, it is known that the EAP20 subunit of ESCRT-II binds to the N-terminal half of the CHMP6 (37, 41). Therefore, we next added CHMP6 to our reconstitutions. To confirm that the entire pathway starting from ALG-2 to CHMP6 is being sequentially recruited onto the membranes, we mixed ALG-2 (dark; 200 nM), ESCRT-I (Cy3; 50 nM), ESCRT-II (dark; 200 nM), and CHMP6 (Atto 488; 400 nM) for 15 min before adding to GUVs. After an additional 15-min incubation, we observed colocalized puncta of CHMP6 with ESCRT-I on the surface of GUVs (Fig. 4*A*, upper row and 4*B*). Omitting either ESCRT-I or ESCRT-II abrogated the membrane recruitment of CHMP6 (Fig. 4*A*, lower row, 4*B*, *C*). Together, this confirmed that the canonical pathway of ESCRT-I/II/III recruitment to membrane can be orchestrated by ALG-2 for membrane repair.

### ALG-2, TSG101, and ALIX Are Corecruited to the Sites of Lysosomal Damage.

**Fig. 5.**
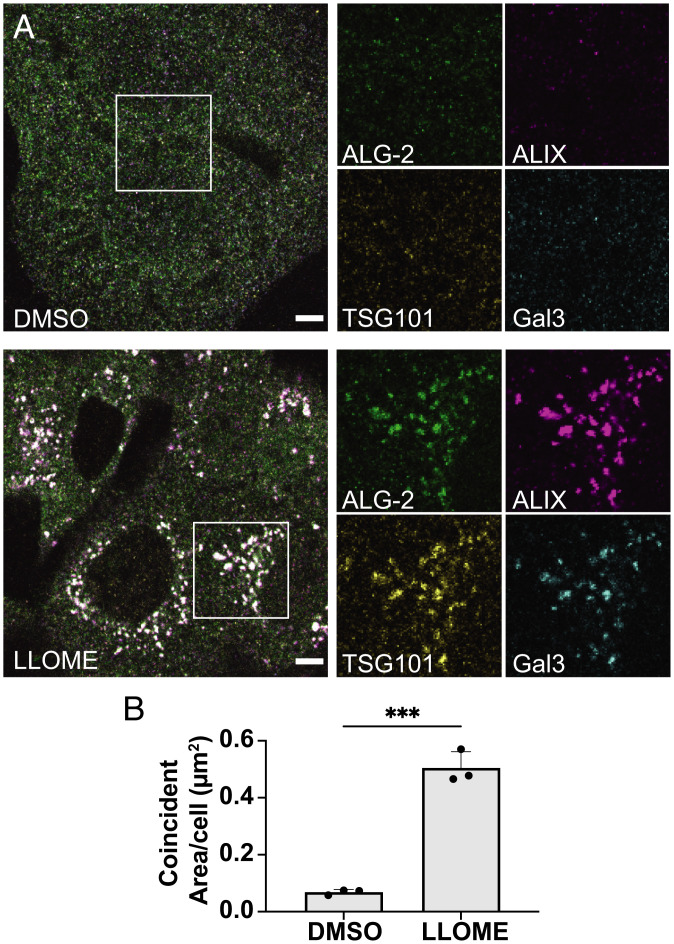
ALIX and the ESCRT-I recruitment factor TSG101 are corecruited with ALG-2 to damaged lysosomes. (*A*) U2OS cells were treated with either DMSO (*Upper row*) or LLOME (*Lower row*) for 15 min and then immunostained for ALG-2 (green), ALIX (magenta), TSG101 (yellow), and Gal-3 (cyan). Relatively large colocalized puncta of ALG-2, ALIX, and TSG101 along with Gal-3 were observed after LLOME treatment. The regions encapsulated within the white boxes have been enlarged and the individual protein channels are shown on the *Right*. Images are representative of five independent replicates. (*B*) The area of overlapping compartments increased dramatically upon LLOME treatment compared to the DMSO control (*n* = 75 cells for DMSO, 64 cells for LLOME). The circles on the bar charts represent independent data points and the data are shown as mean ± SD (vertical line). *P* = 0.0002 (***). (Scale bar, 5 μm.)

Earlier cell-based experiments have shown coaccumulation of ALG-2 with ALIX upon endolysosomal membrane damage (9), as well as coaccumulation of ESCRT-I subunit TSG101 with the ESCRT-II subunit EAP30 (10). The association of ESCRT-I and ALG-2 has not been directly explored, however. Through our in vitro reconstitution experiments, we found ALG-2 to be a viable upstream candidate to recruit the ESCRT-III machinery to the negatively charged membranes via the ALIX and the ESCRT-I/II pathway. Therefore, to validate our findings, we treated U2OS cells with the membrane-permeant lysosomotropic agent L-leucyl-L-leucine methyl ester (LLOME) to induce rapid nanoscale ruptures of endolysosomal membranes (42, 43). Once in the lumen of the lysosomal membrane, LLOME is proteolytically processed into a form that reparably damages the lysosomal membrane. Lysosomal membrane ruptures result in the luminal accumulation of otherwise cytosolic galectin-3 (Gal-3). Therefore, damaged lysosomes can be distinguished from other acidic organelles by virtue of a detectable Gal-3 signal (43). We confirmed that acute LLOME treatment (15-min exposure) resulted in a dramatic shift of diffusely cytoplasmic Gal-3 (Fig. 5*A*, *upper row*) to punctate structures resembling membranous organelles (Fig. 5 *A*, *lower row*). On these damaged lysosomes, immunostaining confirmed the accumulation of ALG-2 and ALIX as previously shown. Additionally, in tune with our GUV-based findings, we observed colocalization of ESCRT-I component TSG101 with ALG-2 at these sites, which also coincided with ALIX (Fig. 5*A*, *Lower*). The statistically significant increase in the coincidence area of ESCRT-I, ALIX, ALG-2, and Gal-3 upon LLOME treatment (Fig. 5*B*) confirms corecruitment of both ALIX and ESCRT-I with ALG-2 on the damaged endolysosomes.

## Discussion

**Fig. 6.**
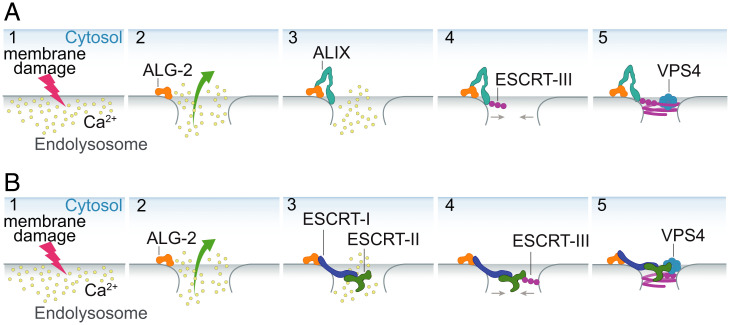
Schematic depicting the Ca^2+^-triggered endolysosomal membrane repair model. A cartoon representation of Ca^2+^-triggered endolysosomal membrane repair by ESCRT-III machinery mediated by ALIX (*A*) or ESCRT-I/II (*B*). The endolysosomal membrane damage leads to Ca^2+^ efflux from the endolysosome into the cytoplasm (*A* and *B*; 1), which leads to the membrane recruitment of the Ca^2+^ binding protein ALG-2 (*A* and *B*; 2). The membrane-recruited ALG-2 leads to downstream recruitment of ESCRT-III proteins via two parallel pathways: the ALIX pathway (*A*; 3 and 4) and the canonical ESCRT-I/II pathway (*B*; 3 and 4). The assembled ESCRT-III machinery forms a higher-order structure that can bring the open ends of the damaged membrane together (*A* and *B*; 5). Finally, remodeling of ESCRT-III filaments by AAA^+^ ATPase VPS4B drives membrane closure and repair.

In this study, through our membrane reconstitution experiments with human ESCRT-III proteins, we substantiated the concept that Ca^2+^ and ALG-2 can be a major trigger for rapid recruitment of ALIX and ESCRT-I to sites of the damaged endolysosomal membrane. A major and unexpected conclusion drawn from our data is that membrane recruitment of ALG-2 can occur in a Ca^2+^-dependent manner without the need for other proteins. The membrane-recruited ALG-2 forms puncta, which could imply the formation of a higher order assembly of ALG-2 on membranes, a topic calling for further exploration. Our observations are consistent with the model proposed by Scheffer et al. for plasma membrane repair (12), suggesting close parallels between the processes at the plasma membrane and lysosomes. Subsequently, we showed the downstream recruitment of the entire ESCRT-III machinery to the membranes with Ca^2+^-activated ALG-2 as the trigger. Most analysis of the ALG-2 pathway in membrane repair has focused on ALIX (9, 11, 12, 44); yet TSG101 is the more important contributor to maintaining cell viability under lysosomal damage conditions (10). Here, we demonstrated that ALG-2 can also recruit ESCRT-I to the membranes in a Ca^2+^-dependent manner. Subsequently, this leads to the recruitment of downstream ESCRT-II complex and CHMP6, which is part of the ESCRT-III machinery. The downstream recruitment of the ESCRT-III machinery in response to Ca^2+^ efflux from the damaged endolysosome could potentially lead to the endolysosomal membrane repair through membrane constriction and scission facilitated by a coherent action of ESCRT-III proteins and AAA^+^ATPase VPS4B (Fig. 6).

Earlier work on plasma membrane repair machinery has proposed ALG-2 to be an important component. In the reconstitution experiments performed by Sonder et al. (17), it was reported that ALG-2 does not bind 10 mol% DOPS membranes on its own. The authors reported that the Ca^2+^-dependent binding of Annexin A7 is necessary to bring ALG-2 to negatively charged membranes (17). However, the amount of negatively charged lipids in membranes used in their study was 10 mol%, which also does not support strong membrane binding in our experiments. Nevertheless, we still see statistically significant membrane binding of ALG-2 at 10% DOPS, albeit the sizes of the puncta are smaller and therefore, at times, difficult to observe (*SI Appendix*, Fig. S5) Sønder et al. (17) used a GFP-tagged ALG-2 construct in their in vitro reconstitution experiments. The presence of a bulky water-soluble tag similar in size to ALG-2 itself in the previous study could have inhibited ALG-2 binding to membranes directly or indirectly through effects on packing or crowding between ALG-2 dimers on the membrane surface. In contrast, we find that ALG-2 labeled with a minimally perturbing small molecule dye does bind to 10 mol% DOPS membranes, although it forms fewer and smaller puncta than at 30 mol%.

Recent cell-based endolysosomal membrane damage studies have shown fast recruitment (within minutes) of ESCRT-III machinery to the membrane damage sites compared to the delayed localization of the initiators of autophagic machinery (9, 10). The arrival of ESCRT-III machinery in response to sites of membrane damage coincided with the restoration of membrane integrity and function (9, 10). While the recruitment of ESCRT-III machinery has been conclusively shown to respond to membrane damage, the specific signals following membrane damage, which trigger ESCRT protein recruitment and the mechanistic roles of the ESCRT machinery in membrane repair, have remained elusive. Our study fills this gap in understanding by showing that ALG-2 is can serve as at least one of the upstream factors to bring downstream ESCRT-III machinery via ALIX and ESCRT-I/II to the damaged endolysosomal membrane. Of course, it is likely that other signals, such as bis(monoacylglycero)phosphate (BMP) (45), annexin A7 (17), or phospho-Rab8A (46), could also contribute to increasing the affinity and specificity of the process.

Cell-based studies of endolysosomal membrane repair have found that silencing of only the ALIX or ESCRT-I/II pathway is insufficient to stall the endolysosomal membrane repair (9, 10, 47). From our reconstitution experiments, we explain this observation by demonstrating that ALG-2 can bind to both ALIX as well as ESCRT-I protein. This can subsequently lead to the recruitment of ESCRT-III machinery by two parallel pathways, as also seen in cytokinesis and other cellular processes in which the ESCRTs function (18). Therefore, our reconstitution experiments explain why silencing of ALIX or TSG101 individually is insufficient to abrogate membrane repair and that silencing of only one gene (out of ALIX and TSG101) can be compensated by the alternate parallel pathway of ESCRT-III recruitment.

The prion-like propagation of molecular aggregates, in particular aggregated tau, is thought to be a prominent mechanism for the spread of misfolded tau in Alzheimer’s disease and frontotemporal degeneration (5). Endolysosomal escape in the receiver cell is a key step in the cell-to-cell spread of aggregated tau (48). A recent CRISPR interference screen-based study showed that knocking down ESCRT components, CHMP6 or CHMP2A together with CHMP2B, increased endolysosomal membrane leakiness and promoted the cytoplasmic entry of tau aggregates (6). Our observation that ALG-2 can recruit ESCRT-III machinery (starting at CHMP6) via the ESCRT-I/II pathway shows how CHMP6 is connected biochemically to the other main players in ESCRT-based lysosomal membrane repair. Our study also offers possibilities for reconstituting in vitro models to study the escape mechanisms of these diseases linked with the endolysosomal escape of protein aggregates.

## Materials and Methods

### Materials.

The lipids 1,2-dioleoyl-sn-glycero-3-phosphocholine (DOPC) and 1,2-dioleoyl-sn-glycero-3-phospho-L-serine (sodium salt) (DOPS) were obtained from Avanti Polar Lipids. DOPE labeled with Atto 647N was purchased from Sigma-Aldrich. HEPES, NaCl, EGTA, and fatty acid-free bovine albumin (BSA) were obtained from Fisher Scientific. All commercial reagents were used without further purification.

### GUV Formation.

GUVs containing DOPC (89.5 or 69.5 mol%), DOPS (10 or 30 mol%), and the lipid fluorophore Atto 647N DOPE (0.5 mol%) were prepared in 270 mOsm sucrose using the polyvinyl alcohol (PVA)-gel hydration-based method as in Weinberger et al. (49). Briefly, lipids were mixed in chloroform at a total concentration of 1 mM. The 40-μL solution of the lipid mixture was spread on a 5% wt/vol PVA film dried on a 25 × 25 mm coverslip (VWR) and then put under vacuum for at least 2 h to form a dry lipid film. The dried lipid film was hydrated with 500 μL of 270 mOsm sucrose solution for 2 h at room temperature to produce GUV dispersion, which was collected and stored in a 1.5-mL microcentrifuge tube.

### Protein Purification.

ALG-2 was purified based on the protocol described in McGourty et al. (50). Briefly, N-terminal 6× His-tagged ALG-2 was expressed in *Escherichia coli* BL21 (DE3) cells in Lysogeny Broth (LB) medium supplemented with kanamycin (50 μg/mL), induced at 0.8 an optical density at 600 nm (OD_600 nm_) with 0.5 mM isopropyl-β-d-thiogalactoside at 37 °C for 3 h. After lysis through tip sonication in lysis buffer (50 mM Tris pH 7.4, 150 mM NaCl, 0.2 mM Tris(2-carboxyethyl)phosphine (TCEP)) the expressed protein was extracted from the supernatant using Ni-nitrilotriacetic acid (Ni-NTA) resin (Qiagen). The resulting eluate from the Ni-NTA resin using lysis buffer supplemented with 250 mM imidazole pH 7.4 was loaded onto the Superdex 75 16/60 column (GE Healthcare) for gel filtration. Subsequently, the resulting solution was purified using anion exchange chromatography using 5 mL HiTrap Q HP (Cytiva). Finally, the eluate was loaded on an equilibrated Superdex 75 16/60 column (GE Healthcare), and the protein purity was assessed using sodium dodecyl sulfate-polyacrylamide gel electrophoresis (SDS-PAGE). The concentration of the purified protein was calculated by measuring the absorbance at 280 nm. Finally, the protein was concentrated at ∼50 μM and stored at −80 °C in small aliquots.

ESCRT-I (TSG101, VPS28, VPS37B, and MVB12A) and full-length ALIX were expressed in HEK293 cells. ESCRT-I had a strep-tagged VPS28 subunit and ALIX was C-terminally strep-tagged and initially purified on StrepTactin Sepharose (IBA), followed by gel filtration chromatography on a Superdex 200 16/60 column (GE Healthcare), in 50 mM Tris pH 7.4, 300 mM NaCl, 0.1 mM TCEP.

ESCRT-II (EAP45, EAP30, and EAP20) was expressed in *E. coli* Rosetta2 (DE3) at 20 °C overnight in LB medium, with an N-terminal tobacco etch virus (TEV)-cleavable 6× His tag and purified on Ni-NTA resin, followed by gel filtration chromatography on a Superdex 200 16/60 column (GE Healthcare), in 50 mM Tris pH 7.4, 300 mM NaCl, 0.1 mM TCEP. The 6× His tag was removed by TEV protease digestion followed by passage over Ni-NTA followed by a final gel filtration chromatography on a Superdex 200 16/60 column (GE Healthcare). The final purified protein was concentrated at ∼10 μM and snap frozen on liquid nitrogen in small aliquots.

ESCRT-III (CHMP6, CHMP4B, CHMP2A, and CHMP3) proteins were purified as described in Carlson and Hurley (31). Briefly, they were expressed individually as an N-terminal TEV-cleavable 6× His-MBP fusion in *E. coli* Rosetta2 (DE3) at 20 °C overnight in LB medium. Cells were lysed by tip sonication, and initial purification was carried out on Ni-NTA resin. In the case of CHMP4B, the 6× His-MBP fusion was further purified on a Superdex 200 16/60 column (GE Healthcare), in 50 mM Tris pH 7.4, 100 mM NaCl, 0.1 mM TCEP. The 6× His-MBP tag was removed by TEV protease digestion at low micromolar concentrations, followed by gel filtration chromatography on a Superdex 75 16/60 column (GE Healthcare), in 50 mM Tris pH 7.4, 100 mM NaCl, 0.1 mM TCEP. CHMP4B is eluted as a monodisperse sample, typically with a concentration of ∼400 nM. Unconcentrated CHMP4B fractions were kept at 4 °C and used within 72 h of purification because CHMP4B formed soluble aggregates over time and freeze–thaw resulted in a loss of material. The other ESCRT-III subunits were purified analogous to CHMP4B, except that the final proteins had higher concentrations (∼20 to 50 μM) and could be snap frozen on liquid nitrogen without aggregation or loss of material. The 6× His-tagged VPS4B was purified similarly to ESCRT-III proteins except an anion exchange step was added between the metal affinity (Ni-NTA) purification and the final gel filtration chromatography step.

Fluorophore labeling was performed overnight at 4 °C using cysteine reactive dyes on engineered N-terminal cysteines (CHMP4B, CHMP2A, CHMP3, and VPS4B), or S118C for CHMP6, or A78C for ALG-2, or on native surface-exposed cysteines (ALIX, ESCRT-I, and ESCRT-II). Specifically, Atto 488 maleimide (Sigma-Aldrich) was used for labeling ALG-2, CHMP4B, ESCRT-II, and CHMP6; sulpho-Cy3 maleimide (Cy3, Fisher Scientific) for labeling ALIX, CHMP3, and ESCRT-I, and Lumidyne 655 maleimide (LD 655, Lumidyne Technologies) for labeling VPS4B. Labeling was performed on the fusion proteins before TEV digest for proteins with a TEV cleavage site. Excess dye was removed by passing the protein–dye mixture through two PD10 columns (Cytiva) sequentially. The final step in every protein purification was gel filtration chromatography so that the monodisperse state of the (labeled) protein could be ensured. Labeling efficiencies were normally 50 to 100%, except for ALG-2, which had a 25% labeling efficiency.

### Reconstitution Reactions and Confocal Microscopy.

The incubation reactions were set up in a microcentrifuge tube at room temperature before transferring to a Lab-Tek II chambered cover glass (Fisher Scientific) for imaging. The imaging chamber was incubated with a 5 mg/mL solution of BSA for 30 min and washed three times with the reaction buffer (25 mM Hepes at pH 7.4, 125 mM NaCl, and 0.2 mM TCEP, 280 mOsm) before transferring the reactants from the microcentrifuge tubes. A total of 15 μL of GUVs was mixed with 120 μL of reaction buffer containing proteins at concentrations stated in *Results*. After a 15-min incubation, images were acquired on a Nikon A1 confocal microscope with a 63× Plan Apochromat 1.4 numerical aperture objective. Three replicates were performed for each experimental condition in different imaging chambers. Identical laser power and gain settings were used for each set of replicates. The reconstitution experiments were performed at room temperature within 48 h of GUV preparation.

### Immunofluorescence.

U2OS osteosarcoma cells were maintained at 37 °C under 5% CO_2_ and propagated using Dulbecco’s Modified Eagle Medium (DMEM) (no. 11965-084, Gibco) supplemented with 10% vol/vol heat-inactivated fetal bovine serum (FBS). Prior to experimentation, cells were seeded into 35-mm glass bottom dishes (no. P35G-1.5-14-C, MatTek) and treated with 1 mM L-leucyl-L-leucine methyl ester (hydrochloride) (LLOME) (no. L7393, Sigma-Aldrich) dissolved in dimethyl sulfoxide (DMSO), or DMSO vehicle control for 15 min. Cells were then fixed in 4% paraformaldehyde (Electron Microscopy Sciences) in PBS for 15 min at room temperature. Cells were then rinsed once with PBS before being permeabilized with 0.02% digitonin (no. BN2006, Thermo Fisher) in PBS for 10 min at room temperature.

Cells were then rinsed once with PBS and blocked with 2% wt/vol BSA in PBS for 30 min at room temperature. Blocked cells were rinsed three times with PBS prior to incubation with primary antibodies diluted in 2% BSA in PBS. ALG-2 was detected using the PDCD6 rabbit polyclonal antibody (no. 12303-1-AP, Thomas Scientific), ALIX, using the PDCD6IP mouse monoclonal antibody (no. MA1-83977, Thermo Fisher), TSG101, using the primary-conjugated mouse monoclonal antibody (no. sc-7964 AF647, Santa Cruz Biotechnology), and Gal-3, using the primary-conjugated mouse monoclonal antibody (no. sc-32790 AF594, Santa Cruz Biotechnology). ALG-2 and ALIX antibodies were diluted at 1:200 with 2% BSA in PBS and incubated with cells for 1 h at room temperature.

To remove nonspecific antibody binding, cells were washed three times with PBS, and antibody binding was probed using secondary-conjugated goat antibodies targeting the species of ALG-2 and ALIX antibodies (no. ab175652, ab150113, Abcam). Secondary antibodies were diluted at 1:500 with 2% BSA in PBS and incubated with cells in the dark for 30 min at room temperature. Cells were washed an additional three times in PBS to remove nonspecific secondary antibodies and subsequently incubated with 10% normal mouse serum (NMS) (015-000-120, Jackson ImmunoResearch) in PBS for 30 min at room temperature to prevent cross-reactivity between secondary antibodies and primary-conjugated antibodies. Cells were then washed three times with PBS and incubated with primary-conjugated antibodies against TSG101 and Gal-3 diluted 1:200 in 10% NMS in PBS for 30 min in the dark at room temperature. Cells were washed a final three times in PBS and imaged immediately.

### Image Analysis.

Custom-made scripts were used to perform puncta recognition analysis in Python. We started by using machine learning to train our model (Yolov5 developed by Ultralytics (https://github.com/ultralytics/yolov5) for GUV recognition. Next, we used this trained model to recognize individual GUVs from confocal image frames with multiple GUVs. For each recognized GUV, we used the corresponding protein channels for puncta recognition. In each protein channel, we performed background subtraction based on the background intensity determined using minimum cross entropy thresholding (threshold_li from scikit-image). Next, we performed Gaussian noise reduction on the background-subtracted images using the fast Nl means denoising algorithm followed by Gaussian blur to get a regularly shaped punctum. Subsequently, we adjusted the image contrast to sparse out the image pixel values (using the equalize bar chart from OpenCV) before generating a binary mask (using threshold_otsu from scikit-image, which minimizes the intragroup pixel value variance). In the foreground pixels, we counted groups of connected pixels that were larger than five pixels as an individual punctum. We calculated the proportion of GUVs with puncta as the ratio of GUVs that have at least one punctum with the total number of recognized GUVs. Finally, for each recognized punctum, we generated a rectangular bounding box. We considered a punctum as colocalized among different protein channels if their bounding boxes overlapped. The proportion of colocalized puncta was calculated as the number of colocalized punctum divided by the total number of puncta in the channel that had the highest number of puncta.

For datasets where no lipid dye was present (Fig. 3), the GUVs were selected manually based on the transmitted light channel. From the selected GUVs, the puncta in the dimmest protein channel (Atto 488, CHMP4B) were detected using the same algorithm described above. These recognized puncta were checked against the other two protein channels for overlap. The remaining process was the same as described for the rest of the datasets.

The representative images in Figs. 1–4 were background subtracted before contrast adjustment for clear puncta depiction. Specifically, the representative figures were thresholded to remove the background fluorescence from the unbound fluorescent protein and clearly depict the puncta observed on the periphery of GUVs. For each representative image, a square area of ∼100 pixels was selected outside the periphery of the GUV in ImageJ. The average pixel value within this selected area was considered the background fluorescence intensity and subtracted from the entire channel. Similar thresholding was done for each (both protein and lipid) channel. Finally, the look-up tables for the background subtracted channels were marginally adjusted to improve the contrast of the puncta on the GUV periphery. This did not affect our quantification analysis, which has been described previously.

For *SI Appendix*, Fig. S5, for comparison between the proportion of GUVs with puncta for 10 vs. 30 mol% DOPS containing GUVs, we changed the selection criterion for puncta in order to reject smaller-sized puncta. Specifically, in the foreground pixels, groups of connected pixels that were larger than 15 pixels were counted as puncta (as opposed to 5 pixels). This was done to differentiate between the smaller-sized puncta observed in GUVs with lower mol% of DOPS (10%).

ImageJ was used for the image analysis of immunofluorescence data. U2OS cells stained via immunofluorescence after treatment with DMSO or LLOME were subject to coincident area quantification. Images of cells were thresholded equally to select regions of interest containing all fluorescent markers. The average pixel area of the intersecting channels was calculated and compared between DMSO- and LLOME-treated samples.

### Statistical Analysis.

Statistical analysis was performed with GraphPad Prism 9.0. The data of the GUV-binding assay were analyzed by Student’s *t* test and one-way analysis of variance. Significance between the two calculated areas was determined using a Student’s two-tailed unpaired *t* test. *P* < 0.05 was considered statistically significant.

## Supplementary Material

Supplementary File

## Data Availability

All study data are included in the article and/or *SI Appendix*.
