# Supplementary material for: In vitro reconstitution of calcium-dependent recruitment of the human ESCRT machinery in lysosomal membrane repair
Source: Proc Natl Acad Sci U S A. 2022 Aug 22;119(35):e2205590119. doi: 10.1073/pnas.2205590119 (PMC9436306; doi:10.1073/pnas.2205590119)
Supplement: Supplementary File [file pnas.2205590119.sapp.pdf]

PNAS

Supplementary Information for

In vitro reconstitution of calcium-dependent recruitment of the human ESCRT machinery in lysosomal membrane repair

Sankalp Shukla[1, 2], Kevin P. Larsen[1, 2], Chenxi Ou[1, 2], Kevin Rose[1, 2], and James H. Hurley[1, 2, 3, *]

Correspondence: James H. Hurley
Email: jimhurley@berkeley.edu

This PDF file includes:

Figures S1 to S5
SI References

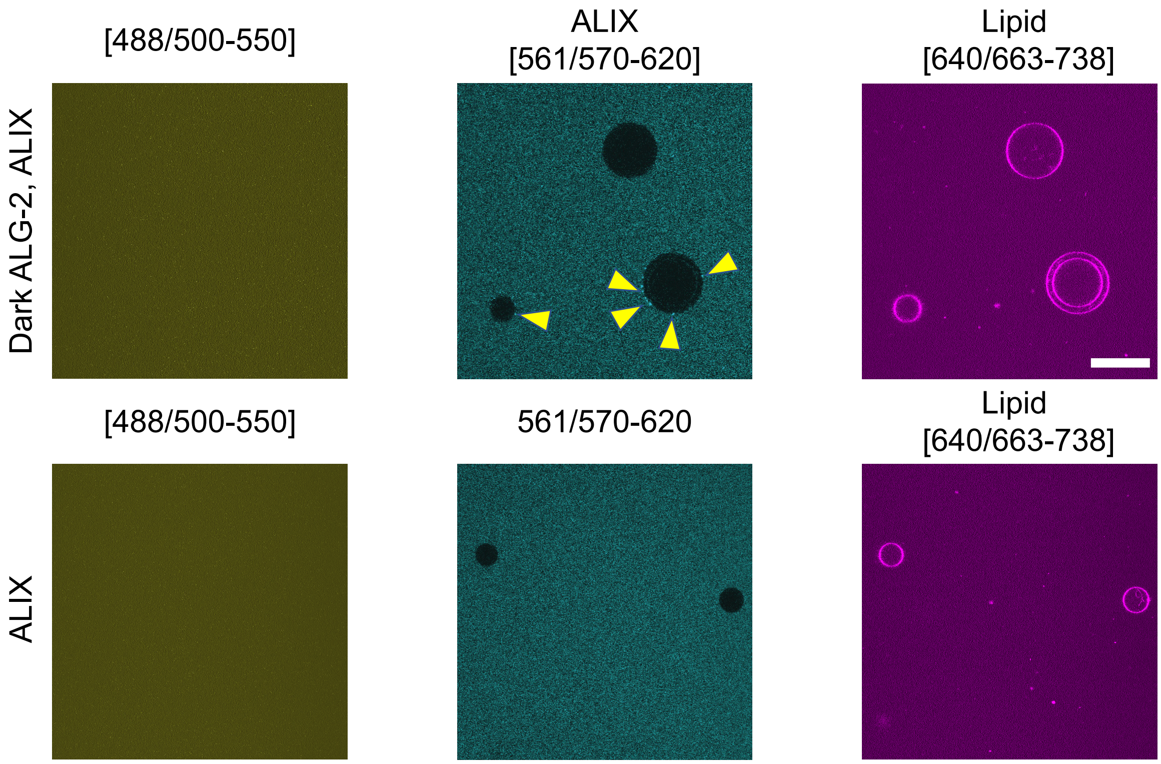

Fig. S1. Unlabeled (dark) ALG-2 recruits ALIX to membranes. The unlabeled ALG-2 can recruit ALIX to membranes similar to the fluorescently labeled W78C ALG-2 (Atto 488; 200 nM) in 30% DOPS containing GUVs. ALIX did not get recruited to GUVs in the absence of ALG-2. Yellow arrows point to ALIX puncta on the periphery of GUVs. Scale bar is 20 μm.

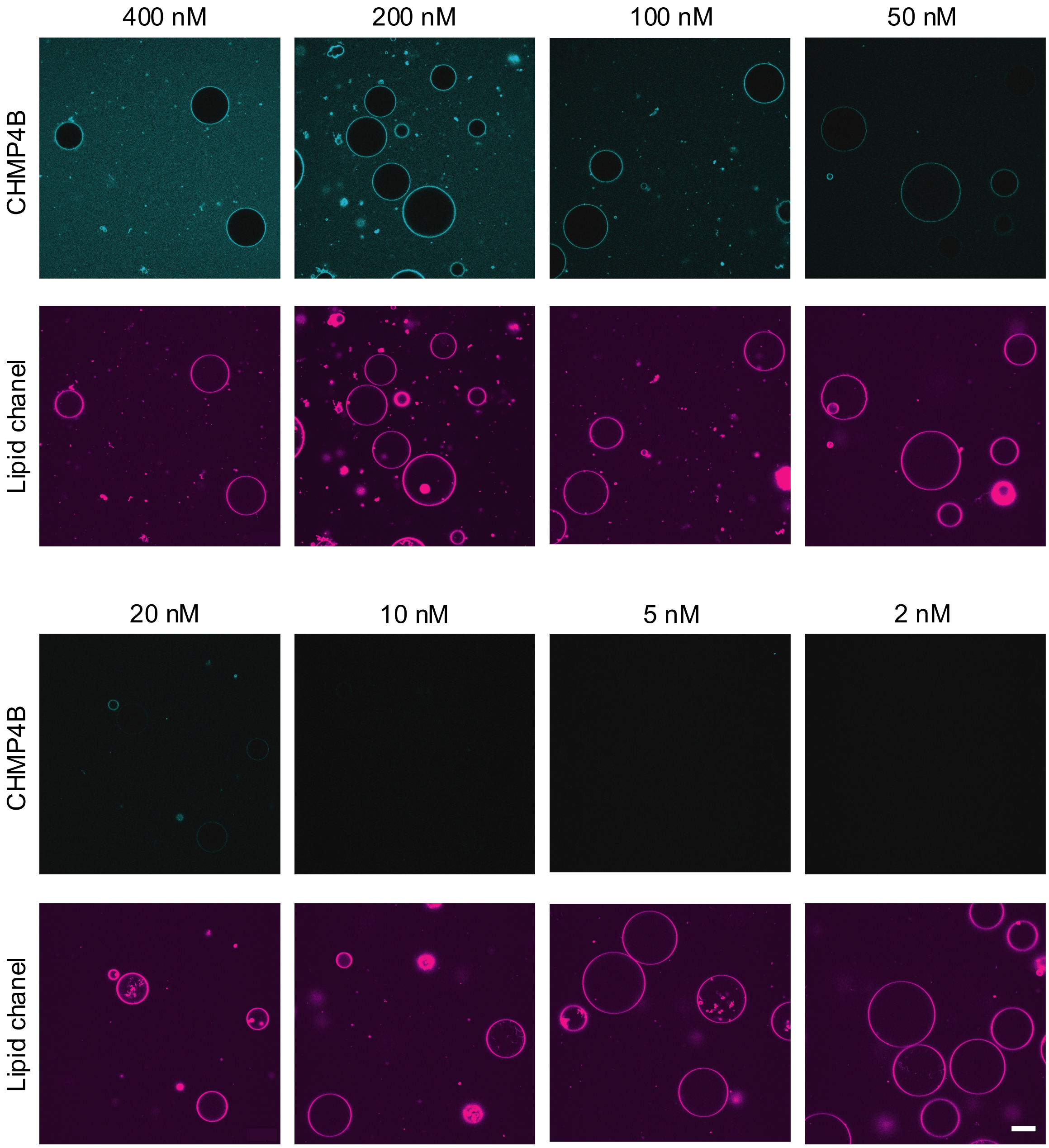

Fig. S2. CHMP4B titration series. CHMP4B can bind to negatively charged membranes at high concentrations. The GUVs containing 30% DOPS were incubated with fluorescently labeled CHMP4B (Atto 488) for 15 minutes at varied concentrations. We observe a decrease in membrane binding of CHMP4B with decrease in concentration. During the time course of our experiments, we did not observe a detectable fluorescence signal of CHMP4B on the membrane periphery below 10 nM. Therefore, we used 10 nM CHMP4B in our reconstitution experiments, so that CHMP4B gets recruited to membranes only in the presence of upstream components. Scale bar is 20 µm.

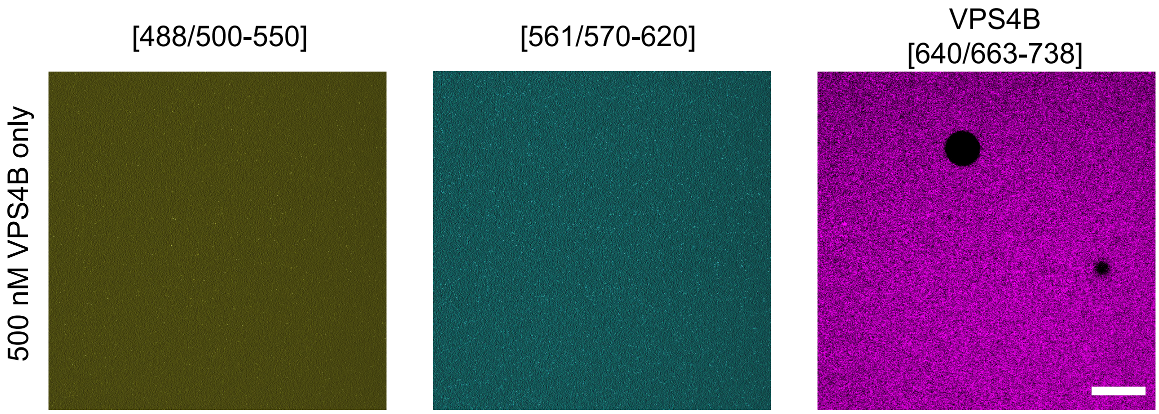

Fig. S3. Absence of membrane recruitment of VPS4B only. GUVS containing 30% DOPS were incubated with 500 nM VPS4B for 15 minutes and imaged. We did not observe membrane binding of fluorescently labeled VPS4B (Lumidyne 655) even at a high concentration of 500 nM in the absence of upstream factors. For our reconstitution experiments we used 100 nM VPS4B. Scale bar is 20 µm.

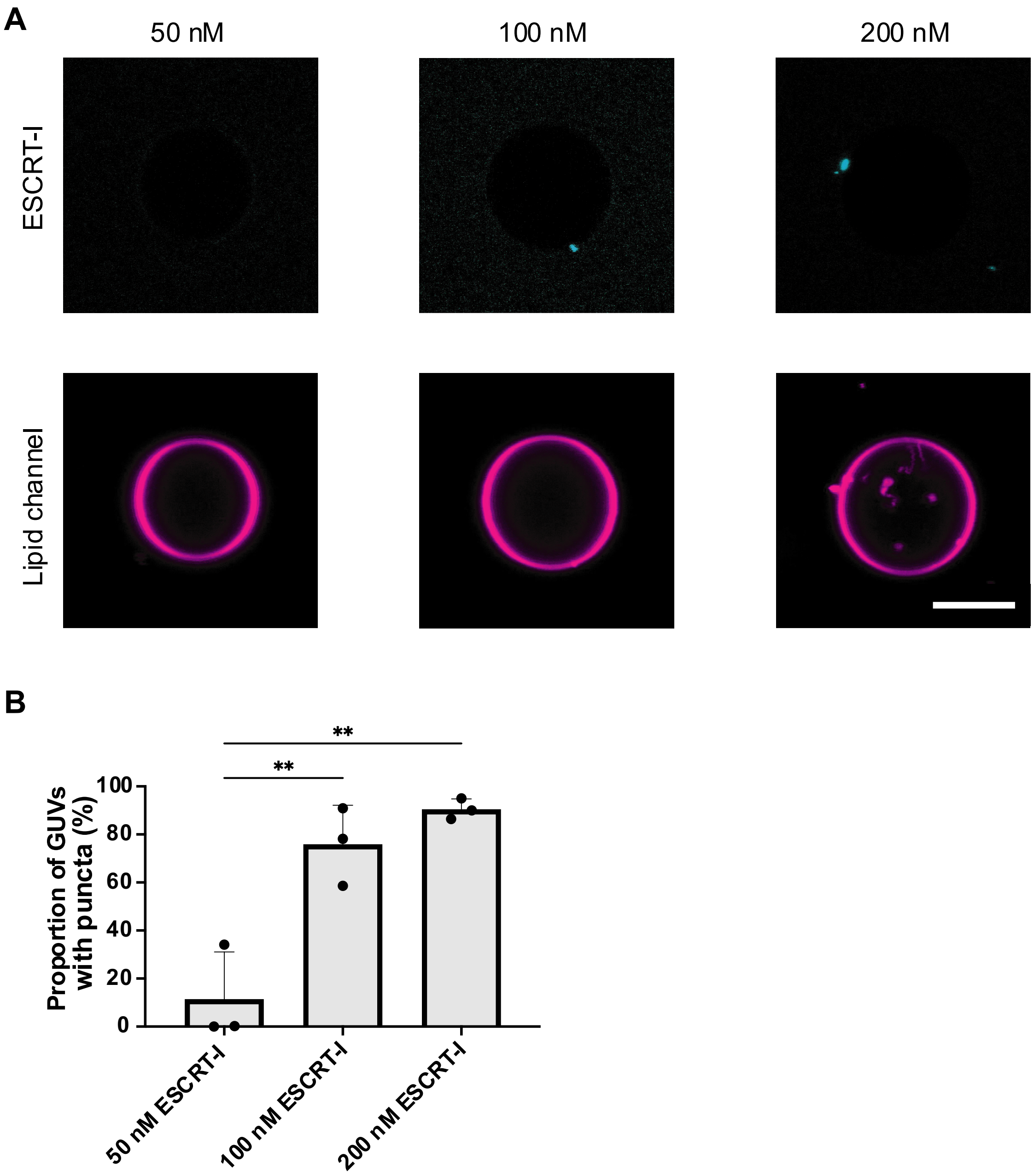

Fig. S4. ESCRT-I titration series. ESCRT-I can weakly bind to negatively charged membranes through its MABP domain (1). We performed a titration series of ESCRT-I with 30% DOPS containing GUVs. A) We observed membrane binding of fluorescently labeled ESCRT-I (Cy3) at concentrations of 100 and 200 nM. We observed significantly lower binding of ESCRT-I to membranes at a concentration of 50 nM. The statistics of the proportion of GUVs with ESCRT-I puncta for 50 nM (N = 299 GUVs), 100 nM (N = 159 GUVs), and 200 nM (N = 109 GUVs) ESCRT-I are shown in B). The dots on the histograms represent independent data points and the data is shown as mean ± SD (vertical line). All results are from three independent experiments. $p \leq 0.0033$ (**). Scale bar is 20 µm.

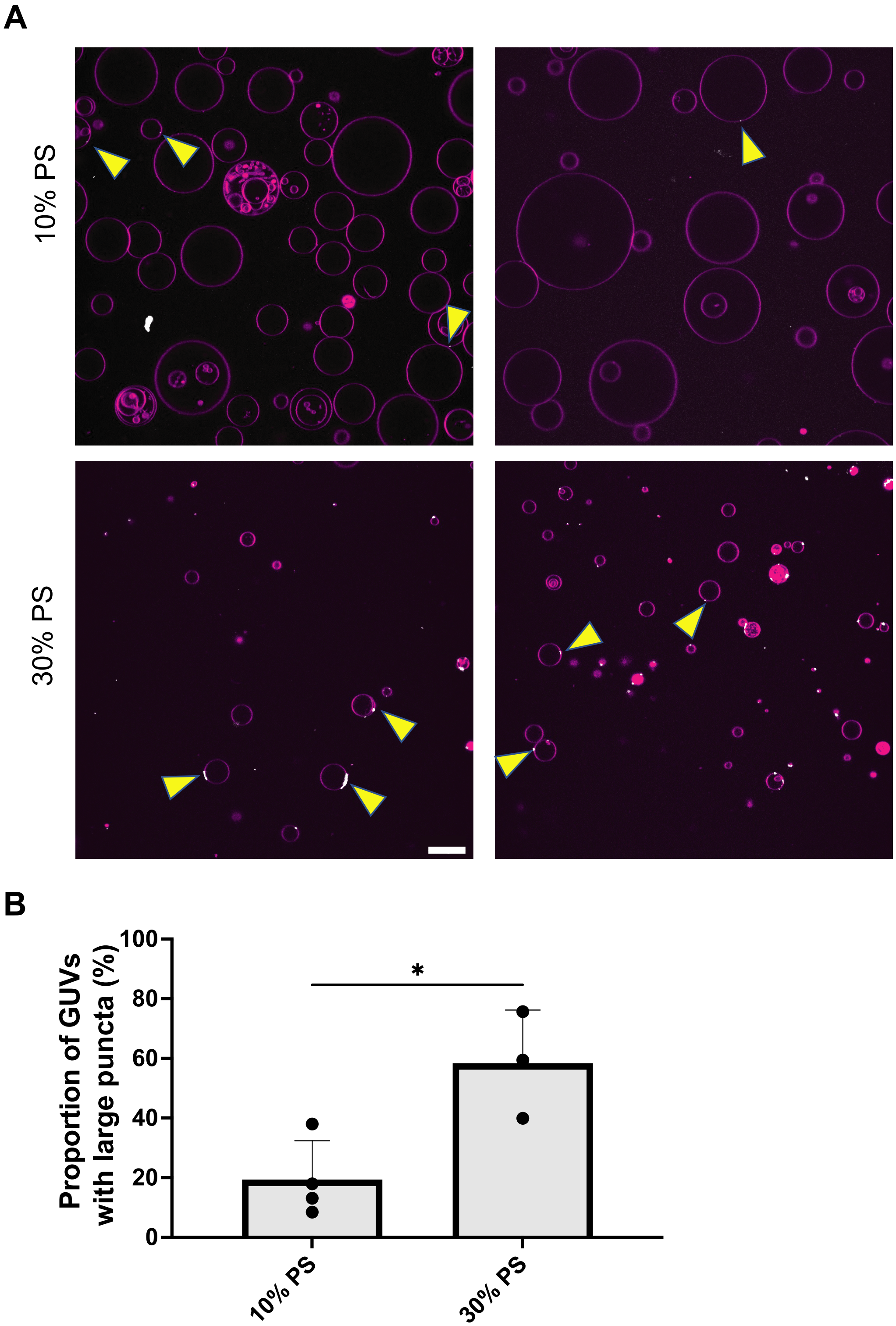

Fig. S5. Negative charge dependent recruitment of ALG-2. A) Fluorescently labeled ALG-2 was incubated with either 10 mol% or 30 mol% PS containing GUVs. ALG-2 puncta were observed in both cases; however, the sizes of the observed puncta were different. Notably, the dimensions of the ALG-2 puncta observed for 10 mol% PS containing GUVs were smaller compared to the puncta observed for 30 mol% containing GUVs. The images show the superimposition of GUV (magenta) channel and ALG-2 (white)

channel. b) The proportion of GUVs that had at least one ALG-2 puncta on their periphery were plotted for 10% PS (N = 520 GUVs) and 30% PS (N = 2064 GUVs) containing GUVs. The puncta detection algorithm was modified to distinguish the smaller sized puncta of ALG-2 on 10 mol% PS containing GUVs to the larger sized puncta on 30 mol% containing GUVs. The details of the modification of the puncta detection algorithm are in the Materials and Method section. The dots on the histograms represent independent data points and the data is shown as mean ± SD (vertical line). All results are from atleast three independent experiments. $p \leq 0.05$ (*). Scale bar is 20 μm.

References

1. E. Boura, J. H. Hurley, Structural basis for membrane targeting by the MVB12-associated β-prism domain of the human ESCRT-I MVB12 subunit. *Proceedings of the National Academy of Sciences* **109**, 1901-1906 (2012).
